# Neuroselling: applying neuroscience to selling for a new business perspective. An analysis on teleshopping advertising

**DOI:** 10.3389/fpsyg.2023.1238879

**Published:** 2023-10-03

**Authors:** Vincenzo Russo, Marco Bilucaglia, Chiara Casiraghi, Simone Chiarelli, Martina Columbano, Alessandro Fici, Fiamma Rivetti, Cristina Rossi, Riccardo Valesi, Margherita Zito

**Affiliations:** ^1^Department of Business, Law, Economics and Consumer Behaviour “Carlo A. Ricciardi”, Università IULM, Milan, Italy; ^2^Behavior and Brain Lab IULM – Neuromarketing Research Center, Università IULM, Milan, Italy; ^3^Department of Management, Università degli Studi di Bergamo, Bergamo, Italy

**Keywords:** neuromarketing, neuroselling, infomercials, EEG, call-to-action, purchase intention

## Abstract

This paper presents an innovative research project that aims to study the emotional factors influencing decision-making elicited by infomercials, a powerful sales technique that uses emotional communication to engage viewers, capture attention, and build trust. Using cutting-edge consumer neuroscience techniques, this study focuses on the identification of the variables that most impact the Call-to-Action and Purchase Intention. Forty participants were selected and divided into two groups, with each group exposed to one of two infomercials (condition A = male seller; condition B = female seller). EEG signals were recorded as well as Eye-tracking data. After the viewing, participants completed a self-report questionnaire. Results show that seller characteristics such as Performance and Trustworthiness, as well as Neurophysiological variables such as Approach-Withdrawal Index, Willingness to Pay, Attention and Engagement, significantly impact the final Call-to-Action, Purchase Intention, and infomercial Likeability responses. Moreover, eye-tracking data revealed that the more time is spent observing crucial areas of the infomercial, the more it will increase our Willingness to Pay and our interest and willingness to approach the infomercial and product. These findings highlight the importance of considering both the Seller attributes and the consumers’ Neurophysiological responses to understand and predict their behaviors in response to marketing stimuli since they all seem to play a crucial role in shaping consumers’ attitudes and purchase intentions. Overall, the study is a significant pilot in the new field of neuroselling, shedding light on crucial emotional aspects of the seller/buyer relationship and providing valuable insights for researchers and marketers.

## 1. Introduction

The study of sales techniques falls within a broad interdisciplinary field that includes economics, finance, logistics, marketing, communication, and psychology (Russo and Gabrielli, 2022). Selling was for a long time classified and explained through formulas and rational models, but the psychological level allows to understand the dynamics occurring in the sales relationships, as psychology, behavioral economics studies and neuroscience suggest that humans are not exclusively rational but are led by affective and emotional processes in the decision-making process. Detecting the elements occurring in the sales-buyers’ relations is therefore crucial for human being understanding. Studying sales techniques and dynamics through psychology and neuroscience, indeed, allows to deepen the human brain functioning and how it can affect human behavior and decision-making process and improve sales dynamics (Cañizares Stay and Cañizares Stay, 2018). Despite the fact that there are few studies on this topic, the opportunity of applying knowledge of the brain and its functioning to managerial processes has enabled us to re-examine these models. The application of neuroscience to selling – *neuroselling* – can be conceptualized as a way of highlighting the role of the human mind in the selling process to optimize sellers’ effectiveness and increase sales (Cañizares Stay and Cañizares Stay, 2018). Despite the fact that neuroselling remains a relatively unexplored field of research, there are some notable works that can be mentioned as pioneers in the field. Studies indicate that neuroselling is becoming increasingly relevant in different areas of selling that is the traditional one, tv, and radio advertising, sales by email, and also door to door selling (Cañizares Stay and Cañizares Stay, 2018). Among the studies which dealt with neuroselling, Sun et al. (2019) used Electroencephalography (EEG) to simultaneously measure brain activity in pairs of subjects during an economic game, revealing that sellers mimic consumers to increase trust. Already in 2008, Tanner et al. (2008) introduced a similar concept, the Chameleon Effect which shows that mimicking the other person leads to better negotiation outcomes. Muñoz et al. (2019) implemented EEG to determine the most attention-grabbing sales techniques, yielding interesting insights for adjusting sales techniques to reach the “neurocognitive pathways of attention.” Neurophysiological indices have also been measured in salespeople (Randolph et al., 2013), revealing different neuronal activations depending on their experience: while experienced sellers record higher relaxation levels, sales managers record higher cognitive load (higher prefrontal activation). From these few but promising studies emerges the importance of further investigation on selling, to better analyze the emotional dynamics and bring insights aimed at improving salespeople’s performance.

Neurosciences, therefore, can be applied to several areas beyond management and economics (Ma and Wang, 2006). In the selling area it allows to understand the brain answer to several stimuli to capture which strategy can lead to the buying decision (Russo and Gabrielli, 2022). Studies on consumer behavior and decision-making processes, suggest that assessment based on the registration of neurophysiological parameters can show reliable results because they lack the mediation of cognitive processes (Poels and DeWitte, 2006; Missaglia et al., 2017). When applied to marketing issues, neuroscience can capture and discover what is happening in the brain in response to several advertising or communication stimuli and, in turn, can lead to the understanding of buying process (Ciceri et al., 2019; Russo et al., 2020). In that regard, this method has proven to be able to predict the purchase behavior and preferences of the population by generalizing the results obtained from the experimental sample (Dmochowski et al., 2014; Barnett and Cerf, 2017; Christoforou et al., 2017; Zito et al., 2021). Therefore, neuroscience techniques, in their attempt to interpret reality based on cognitive schemes and experienced emotions and on the detection of decision-making process, can be applied in any communication exchange activating a detectable and observable reaction (Plassmann et al., 2015; Bazzani et al., 2020). Moreover, studies suggest that the neural science applied to financial decision-making, becomes a science overcoming the only psychological aspects, even deepening the mind of the decision-maker, trying to understand realistic functioning of the decision instances to explain a larger area of the individual economic behaviors (Vasile and Sebastian, 2007).

An important element that can affect the decision process and the relationships between subjects, thus affecting the emotional side and, consequently, the selling-buying process, is trust. Trust has been defined for a long time as a condition in interpersonal selling, but the possibility to personalize the service or the product even on the internet or non-store direct selling is also important for the trust occurrence (Young and Albaum, 2003). According to these studies, in the marketing area the possibility to have trust is psychologically linked to the management of uncertainty and, in the specific case of this study, where the direct relationship is not tangible, but credibility and trust are so central, the seller’s behavior and style is crucial. On this issue, studies suggest that the seller’s communication style differs by gender (McQuiston and Morris, 2009), and communication style affects attraction. Indeed, the ability of sellers to adapt their communication style to customers is a crucial factor (Alavi et al., 2019), as it has been observed that people decode persuasive messages according to different styles that assign importance to dissimilar aspects of communication. For instance, these styles include the well-known division in “selective-processors” and “comprehensive-processors” (Meyers-Levy and Loken, 2015). Similarly, it has been shown that socio-demographic properties such as gender (Levy and Sharma, 1994) and relational variables like trust are also relevant factors in selling (Palmatier et al., 2006). Due to these considerations, it appears necessary to investigate how the seller’s gender, communication style, and trust are intertwined with the subjects’ emotional reactions. Emotional reactions in the seller/buyer relationship have been understudied due to the challenge of measuring emotions (Young and Albaum, 2003). However, consumer neuroscience can fill this gap by examining both conscious and unconscious decision-making processes that influence behavior (Hazeldine, 2014).

In fact, measuring an emotional reaction to an advertisement with these techniques allows for real-time detection of the most spontaneous aspects of consumers’ emotional reactions, through variation in body parameters that reflect emotional activation (Damasio, 1994; Vitouch, 1997; Bechara et al., 2000; Larsen et al., 2003; Poels and DeWitte, 2006; Posner, 2006; Kenning et al., 2007). For example, a study conducted using neuromarketing techniques, showed that listening to radio advertisement subsequently leads to spending more time and more involvement in watching the advertised brand or the same spot on TV (Russo et al., 2020). Neuromarketing techniques have proven useful in choosing the most effective commercial in terms of liking and ability to activate mnemonic processes (Russo et al., 2022). For instance, in Unicef commercials, the detection of emotional responses through neuromarketing tools captured both emotional intensity and cognitive engagement. The modification of the commercials led to a 35 percent increase in donations (Zito et al., 2021). For this reason, the use of neuromarketing techniques over the years has had increasing interest (Lee et al., 2007).

According to literature on neuroselling and its relevance on several areas, sales techniques have also become subject of interest outside real-world contexts, demonstrating that they can exert a persuasive effect even within mediated relationships, as in the case of teleshopping (Cañizares Stay and Cañizares Stay, 2018). Teleshopping, which consists of the purchase of products or services through the medium of television, has proved to be one of the fastest growing forms of marketing in the last 20 years (Bouhlel, 2019) and is a privileged place where persuasive techniques are implemented. As numerous studies have shown, the ability of media sellers to establish a relationship with consumers is determined by psychological phenomena called parasocial interaction (PSI) and parasocial relationship (PSR). PSI, on the one hand, refers to the reaction a media user has toward the performer, such that the user perceives the performer as a relational partner. PSR, on the other hand, is inherent in a lasting relationship that the user establishes with the media performer (Dibble et al., 2016). Both of these constructs are favored by the behavioral styles enacted by the TV performer. In reference to this, the literature on teleshopping has identified a number of persuasion techniques used by salespeople that relate to both the rhetorical styles and psychological factors of the consumer. Fritchie and Johnson (2003) verified that teleshopping programs regularly used the 6 sales strategies identified by Robert Cialdini (1993) by measuring their prevalence in 23 programs. The results showed that 35.5% of the channels adopted the technique of social proof, 28.4% scarcity, 18.4% authority, 9.1% commitment and consistency, 6.1% liking, and 2.0% reciprocity. Cortese and Rubin (2010) found that people who make more planned purchases tend to look at the commercial aspects of the medium but did not perceive an interpersonal connection with the presenters. In the same study, those who purchased more items were motivated to watch the commercial aspects of the TV medium. Kline (2005) has found that teleshopping programs resort to a number of rhetorical styles aimed at influencing the viewer’s decision-making process. In particular, scholars have identified 5 classes of rhetorical styles called “construction of trustworthy relationships,” “seeking consensus through inquiry and advocacy,” “creating a believable reality for product worth,” “staging impressive messages for viewers,” and “facilitating teleshopper commitment.” Trusting relationships are created by the salesperson either by communicating praise, inclusion, and interest to the viewers, or by verbally expressing their own feelings and aspects of their personal life. Seeking consensus was achieved by stimulating the audience’s needs and desires to which the seller provided the solution through the product. Creating a believable reality for product worth was ensured by providing factual information on product properties, such as materials, dimensions, and the production process. Impressive messages relied on emphasizing the pronunciation of words, using colorful terms and descriptions that dramatized product features. Teleshoppers’ commitment to purchase was stimulated through certain heuristics highlighting the exceptionality of the low price and the scarcity of the product. According to scholars, sellers tend to combine several of these communicative styles simultaneously, a concept that has been called “lamination” (Kline, 2005). The salesperson, as a persuader, has to adopt effective persuasive strategies by adapting to the particular needs of the target customer. He has to get to know his customer and, prioritizing the customer’s interests, design customized strategies for approaching, attracting attention, and satisfying needs. Numerous studies have shown that trustworthiness is directly related to persuasion and message effectiveness. A review by Pornpitakpan (2004) on source credibility and its effects on persuasion indicates the superiority of a high-credibility source over a low-credibility one: the more reliable a communicator is, the more effective his or her opinion will be to the receiver of the message. In addition, the results of one study confirmed that the perceived credibility of the source indirectly affects purchase intention (Muda and Hamzah, 2021). Babin et al. (2004) studied how customers form ethical perceptions while interacting with a salesperson and the consequent effects of these perceptions on customer emotions and purchase intentions. They found that moral fairness (a customer’s perception of being treated fairly by a salesperson) promotes positive emotions and inhibits negative emotions. Moreover, they observed how positive emotions increase customers’ future purchase intentions while negative emotions reduce them. One of the main tools for the salesperson in this process is language. For Verschueren (1999), the use of language must consist of the continuous making of language choices. According to this author, choices are mainly based on 3 characteristics: variability, negotiability, and adaptability (Verschueren, 1999). Variability allows the user to make choices at all levels of language structure. Negotiability is responsible for highly flexible principles and strategies underlying choices. Lastly, adaptability allows humans to make negotiable language choices and approach points of communicative need satisfaction. Many of these characteristics were found in a study analyzing TV shopping (Huang, 2019). The results of another study analyzing the relationship between TV shopping and antecedent motivations in media use through the uses-and-gratifications theory (U&G, a user-centered approach that focuses on how people use media for their own personal uses and gratification) perspective, suggest that TV viewers watch TV shopping because they are motivated by a convenient, cheap and fast way to shop, learn about products and finally, they want to be entertained (Cortese and Rubin, 2010). Finally, other persuasive levers are related to the upward social mobility ensured by the product (e.g., high-end articles) (Cook, 2000), the pressure exerted by the reduced time within which to decide on a purchase (Auter and Moore, 1993), and the possibility to easily return the product and be refunded (Hariramani, 2018). At the level of psychological variables, just as in real sales contexts, in the case of mediated relationships, trust has been shown to be a facilitating factor in the purchase decision (Bouhlel, 2019). In addition, customer satisfaction showed to be correlated with perceived value, convenience, and product variety (Sahai et al., 2020), although only convenience has been shown to be predictive. Currently, it is important to explore what determines communication effectiveness in terms of sales. In fact, most of the research conducted on the communication style of salespeople dates back to the 1980s and 1990s. For this reason, many of these studies do not take into account the influence that new technologies can have on sales. Indeed, there has been a shift from seller–buyer communication to computer-buyer communication (Chaganti et al., 2017). The availability of product details, reviews, customer satisfaction ratings, and so on, are so comprehensive that they have the potential to trigger a decision on the part of the customer, thus minimizing the communication needs in the seller–buyer interaction (Chaganti et al., 2017).

## 2. Objectives

The main objective of this project is to study infomercials, an effective sales technique that uses emotional communication to engage viewers, capture attention, and build trust (Fritchie and Johnson, 2003). In particular, we want to investigate the relationship between the seller attributes (e.g., gender, performance, perceived trustworthiness, manipulatory communication) and neurophysiological indicators of approach, willingness to pay, engagement and attention in determining Call-to-Action and Purchase Intention responses and the overall infomercial Likeability.

This project emerges as novel research, bridging neuroscience and sales research by examining emotions in purchase decision-making and answering the following research questions:

Q1: Can neuroscientific tools enhance our understanding of selling communication techniques?

Q2: What neurophysiological variables mostly impact Call-to-Action, Purchase Intention and overall Infomercial Likeability?

Q3: What neurophysiological variables are mostly associated with the Time Spent on the areas of interest crucial to infomercial communication?

## 3. Materials and methods

### 3.1. Stimuli

Two videos with a duration of 1.31 min were selected as stimuli for the study corresponding to two infomercials by Eminflex (a brand that sells mattresses). Both videos promoted identical products and offers, presenting a congruent narrative structure. Nonetheless, one video featured a male seller, while the other showcased a female seller. Both videos are accessible online via the following links: https://www.youtube.com/watch?v=JLvAwnRN7Tw (male seller), https://www.youtube.com/watch?v=SYDZurn0ZVU (female seller). The video narration was designed in the following way: the salesperson welcomed the viewer, then presented the product, its offer, and finally the call to action. This homogeneity between stimuli allowed to compare the possible effects of gender. Prior to the experiment, participants were kept unaware of the content of the infomercials in order to prevent this factor from influencing their responses during the study.

### 3.2. Sample

The research was conducted on 40 respondents, equally distributed by gender (20 females) and aged 20–55 years (*M* = 36.7; SD = 14.4,). The total sample was then divided into two experimental groups consisting of 20 subjects (10 females) each and assigned to two experimental conditions:

– Condition A in which the infomercial was administered with the male seller
– Condition B in which the infomercial presented had the female seller as the presenter.

A 2-way ANOVA considering the age as dependent variable did not show any significant effects for both the gender [*F*(36,1) = 0.009, *p* = 0.925] and group [F(36,1)==0.03, *p* = 0.958] factors, as for the gender*group interaction [*F*(36,1) = 0.005, *p* = 0.942]. The study protocol was approved by the ethical committee of IULM University and informed written consent was obtained from each participant before starting the experiment.

### 3.3. Experimental procedure

Once informed consent was signed, participants entered the experimental room and sat on a comfortable chair in front of a 23.8″ monitor at a distance of 60–70 cm. The sensors for neurophysiological measures – Electroencephalography (EEG) – were then attached to the subjects, and then the eye tracker was calibrated.

A between-subjects research design was used for the study: each of the two samples was administered one of two infomercials and 5 commercials used as distractors, with the aim of simulating a typical commercial break. All videos (distractor commercials and target infomercials) were presented in random order so as to avoid any bias attributable to a position effect. Respondents were asked to freely observe the stimuli presented on the screen, and to try to move as little as possible throughout the duration of the protocol to minimize motion artifacts. Before the video stimuli were administered, participants performed two relaxation phases lasting 5 min each, with the goal of recording baselines. The first (EYC) was performed with eyes closed, the second (BSL) with eyes open while observing a black screen with a white dot in the center. Immediately after the presentation of the target video, the infomercial, for both conditions, A and B, a question was administered to collect data on the call to action. Finally, at the end of the experimental protocol the subjects were administered a self-report questionnaire with the aim of collecting data on the respondents’ rational experience.

### 3.4. Instruments

EEG and SC signals were recorded via NeoRec v.1.5 (Medical Computer System) software, while the eye-tracker signal was recorded via iMotion software (iMotions, S/A). All stimuli were presented via iMotions. Synchronization between NeoRec v.1.5 and iMotions was performed via the ESB - EEG Synchronization Box (Bilucaglia et al., 2020) from a signal generated by the USB-TTL adapter SH-U35A (DSD Tech.) at the beginning of the experiment.

#### 3.4.1. Electroencephalography

Electroencephalography (EEG) signal was recorded through the NVX 52 device (Medical Computer System), at a sampling rate of 2000 Hz and a resolution of 24 bits. Forty Ag/AgCl electrodes were placed at locations Fp1, FpZ, Fp2, F7, F3, Fz, F4, F8, Ft7, Fc3, Fcz, Fc4, Ft8, T3, C3, Cz, C4, T4, Tp7, Cp3, Cpz, Cp4, Tp8, A1, T5, P3, Pz, P4, T6, A2, P5, P6, Po7, Po3, Poz, Po4, Po8, O1, Oz, O2, according to the international 10–10 system (Jurcak et al., 2007). The ground electrode was placed on the left mastoid, and the reference was calculated from the auricular electrodes A1-A2: left channels were referred to A1, right channels to A2, and middle channels to the mean (A1 + A2)/2. Before electrode placement, the skin was abraded with Nu Prep gel (Spes Medica, S.r.l). Then, Neurgel gel (Spes Medica, S.r.l.) was used to lower the electrode impedance below 20kΩ.

The EEG signal was processed using Matlab (The Mathworks, Inc.) and the EEGLab toolbox (Delorme and Makeig, 2004): first, it was re-referenced to CAR (Common Average Reference), bandpass filtered between 2 and 48 Hz, and notch filtered at 50 and 100 Hz. An Independent Component Analysis (ICA) was performed using the FastICA algorithm. Once obtained (Independent Components) were categorized by ICLabel, a neural network classifier (Pion-Tonachini et al., 2019): ICs identified as not “brain” with probability greater than 0.7 were labeled as “artifacts.” “Artifact” ICs were removed, and the signal was back-projected into sensor space. ICA, classification, removal of ICs, and back-projection were performed a second time to eliminate residual artifacts.

For each subject, the Individual Alpha Frequency (IAF) was estimated as the center of gravity within the [7.5 Hz; 12.5 Hz] band of the Power Spectral Density (PSD) averaged across all the occipital channels (Klimesch, 1999). The PSD was estimated within the EYC baseline, following the Welch’s approach based on a 1 s-long hamming window and 50% of overlapping. Theta(θ), Alpha (α), Beta (β) and Gamma (γ) bands were defined, according to the estimated IAF, as: θ = [IAF-6; IAF-6], α = [IAF-2; IAF + 2], β = [IAF + 2; IAF + 16] and γ = [IAF + 16; IAF + 25] (Borghini et al., 2019).

The following EEG metrics were finally calculated to assess neurocognitive reaction to target stimuli:

– Approach Withdrawal Index (AWI), computed as the difference of α powers between right and left prefrontal channels. It is a well-known measure of motivational tendency: positive AWI values are related to a cognitive approach toward the stimulus, while negative ones are related to cognitive withdrawal (Davidson et al., 1990).
– Willingness to Pay Index (WPI), computed as the difference of γ powers between right and left prefrontal channels. It was derived from a study by Ramsøy et al. (2018) that positively correlated it to the respondents’ willingness to pay.
– Beta over Alpha plus Theta Ratio (BATR), computed as the ratio between β group powers and the sum of α and θ powers of all of the EEG channels. It has been proposed as a measure of Cognitive Engagement (Freeman et al., 1999).
– Beta over Alpha Ratio index (BAR), computed as the ratio between the α and β powers of all the EEG channels. It has been proposed as a measure of emotional arousal (McQuiston and Morris, 2009).

These metrics were calculated using the spectrogram approach and consisted in time-signals with 0.5 s of temporal resolution (Gabrielli et al., 2020). After the metric computation, a z-scored transform according to the mean value and the standard deviation computed within the BSL baseline was first applied; then, the transformed signals were epoched according to the commercial’s onset and offset. The epoched signal was thus segmented into three parts that were found homogeneous in both the commercials: an initial part, where the seller gives a gentle introduction to both the company and the product; a central part, where the seller details the unique features of the product, the product bundling and the payment options; a final part, that consists in the call-to-action. The signal was finally averaged within the segments, to get, for each metric, three mean values named <X > InitialSection, <X > CentralSection, and < X > FinalSection, where <X > is the metric name (i.e., AWI, WPI, BATR or BAR). This splitting procedure allows the assessment of the individual contributions of the narrative parts on the Call-to-Action, Purchase Intention, and infomercial Likeability, avoiding any masking effect due to a temporal average of the overall commercial (Russo et al., 2022).

#### 3.4.2. Eye-tracker

Eye-tracker data were acquired through the EyeTracker ProSpectrum bar (Tobii LLC), with a sampling rate of 60 Hz. Eye-tracker data were then analyzed through iMotions software to identify gaze fixations on the target stimuli. Specifically, 3 Areas Of Interest (AOIs) were selected: the brand Logo, the Discount, and the Price. From each AOI, Time Spent metrics were extracted as a measure of visibility and saliency (Russo et al., 2021).

#### 3.4.3. Questionnaire

At the end of the video stimuli administration, subjects had to fill a self-report questionnaire to further investigate their rational evaluation of the target stimuli. Specifically, the questionnaire included 6-point Likert scales (1 = nothing, 6 = very much) aimed at evaluating different variables. More specifically:

– *Purchase Intention* has been detected through 1 *ad hoc* item created to measure the degree of intention to buy of the televiewer. The item is: “*How willing are you to buy the product promoted by teleshopping?*”
– *Call to Action* has been detected through 1 *ad hoc* item created to measure the degree of intention to call the sellers to have more information. The item is: “*Indicate your intention to call the number indicated to receive more information*.”
– *Infomercial Likeability* has been detected through 1 *ad hoc* item created to measure the degree of appreciation of teleshopping. The item is: “*How much did you like the viewed teleshopping?*”
– *Offer Perceived Convenience* has been detected through 1 *ad hoc* item created to measure the perception of convenience of the product promoted by the seller. The item is: “*How convenient do you consider the offer promoted by teleshopping?”*
– *Manipulatory communication* has been detected through 1 item based on the work by Dion and Notarantonio (1992) to measure the perception of manipulation adopted by the seller. The item is: “*I feel that the seller’s communication style is manipulatory.”*
– *Seller Performance* has been detected through 7 items based on the work by Dion and Notarantonio (1992). An example item is “*The seller makes the product credible*.” To assess psychometric properties a factorial analysis was performed founding a unique factor with an explained variance of 67%. Crobach’s alpha for this measure is very satisfactory: *α* = 0.92.
– *Affective Trustworthiness* has been assessed through 4 items from Akrout et al. (2016). An example item is “*I am sure that he will always make me his best offer*.” To assess psychometric properties a factorial analysis was performed founding a unique factor with an explained variance of 68%. Crobach’s alpha for this measure is very satisfactory: *α* = 0.94.

#### 3.4.4. Statistical analyses

Analyses were conducted with the statistical software Jamovi (version 2.3) to identify differences in responses based on the gender of the seller (male or female), communication skills of the salesperson, age of the respondents, and EEG metrics. Prior to this comparison, a preliminary analysis was conducted to highlight differences in cognitive load and any differences related to respondents’ gender: no differences were revealed for these two variables. The recorded data were then analyzed using the following statistical tests:

– 3 Linear regression models were run to investigate the variables (both questionnaire variables and Neurophysiological metrics such as AWI, WPI, BATR, and BAR indexes) that most predicted Call-to-Action, Purchase Intention, and infomercial Likeability.
– a Pearson’s linear correlation to see relationships between Time spent on certain AOIs crucial to communication and EEG metrics (AWI, WPI, BATR, and BAR indexes).

Before constructing the three regression models, a preliminary assessment of the assumptions of Normality and Heteroskedasticity was conducted. Due to the relatively modest sample size (N = 40), the Shapiro–Wilk test was selected to assess Normality, while the Breusch-Pagan test was employed to evaluate Heteroskedasticity. Notably, all assumptions were found to be upheld across all three models.

Moreover, to assess the psychometric characteristics of the questionnaire measures, factorial analysis, and Cronbach’s alphas were performed with SPSS 27.

## 4. Results

### 4.1. Linear regression models

A linear regression model was conducted to investigate which of the variables investigated had a significant impact on Call-to-Action (Table 1). The regression model accounts for 60% of the variance (Adjusted *R*^2^ = 0.603, *F* = 3.61, *p* = 0.011) (Table 1A). The regression model suggests that Seller Performance (*β*st = 0.5017, *p* = 0.038) and Neurophysiological variables, specifically the WPI recorded in the Initial (*β*st = 15.597, *p* = 0.047) and Central (*β*st = −34.643, *p* = 0.050) sections of the spot, are involved in significantly explaining the final Call-to-Action (Table 1B). Normality and Heteroskedasticity assumptions were checked and found to be met (Table 1C).

**Table 1:** Linear regression model with Call-to-Action as dependent variable.

(A) Model fit measures								
					Overall model test			
Model	*R*	*R* ^2^	Adjusted *R*^2^	AIC	*F*	df1	df2	*p*
1	0.913	0.833	0.603	87.0	3.61	18	13	0.011

(B) Model Coefficients – Call-to-Action					
Predictor	Estimate	SE	*t*	*p*	Stand. estimate
Intercept	−11.122	0.7921	−140.416	0.182	
Age	0.0204	0.0171	118.976	0.254	0.2298
Seller gender	0.5196	0.3710	140.051	0.183	0.4138
Affective trustworthiness	0.1697	0.2546	0.66635	0.516	0.1755
Seller performance	0.6776	0.2964	228.620	0.038*	0.5017
Manipulatory communication	0.0505	0.1223	0.41318	0.686	0.0697
AWI_InitialSection	0.2105	0.1505	139.847	0.184	0.9507
AWI_CentralSection	−0.3083	0.2331	−132.258	0.207	−10.271
AWI_FinalSection	0.0744	0.0889	0.83666	0.417	0.2294
WPI_CentralSection	−10.607	0.4952	−214.201	0.050*	−34.643
WPI_InitialSection	0.3344	0.1539	217.237	0.047*	15.597
WPI_FinalSection	0.5954	0.2881	206.698	0.058	20.950
BAR_InitialSection	−107.197	78.862	−135.931	0.196	−0.4176
BAR_CentralSection	−21.750	233.862	−0.09300	0.927	−0.1465
BAR_FinalSection	41.096	258.657	0.15888	0.876	0.2342
BATR_InitialSection	56.836	43.284	131.311	0.210	0.3866
BATR_CentraleSection	−29.066	300.341	−0.09678	0.924	−0.2285
BATR_FinalSection	−0.2399	317.404	−0.00756	0.994	−0.0171

(C) Assumption checks			
Test type		Statistic	*p*
Normality test	Shapiro–Wilk	0.943	0.089
Heteroskedasticity test	Breusch-Pagan	9.28	0.901

**(A)** Model fit measures table, **(B)** Model coefficients table. *p < 0.05, **(C)** Assumption checks table.

A second linear regression model was conducted to investigate which of the variables investigated had a significant impact on Purchase Intention (Table 2). The regression model accounts for 64% of the variance (Adjusted *R*^2^ = 0.636, *F* = 4.01, *p* = 0.007) (Table 2A). The regression model suggests that the Affective Trustworthiness (*β*st = 0.6135, *p* = 0.041) and Neurophysiological variables, specifically the AWI (βst = 1.4484, *p* = 0.042) and BAR (*β*st = −0.7175, *p* = 0.030) recorded in the Initial section of the spot, are involved in significantly explaining the final Purchase Intention response (Table 2B). Normality and Heteroskedasticity assumptions were checked and found to be met (Table 2C).

**Table 2:** Linear regression model with Purchase Intention as dependent variable.

(A) Model fit measures								
					Overall model test			
Model	*R*	*R* ^2^	Adjusted *R*^2^	AIC	*F*	df1	df2	*p*
1	0.921	0.847	0.636	78.9	4.01	18	13	0.007

(B) Model coefficients – purchase intention					
Predictor	Estimate	SE	*t*	*p*	Stand. estimate
Intercept	0.1263	0.7382	0.1710	0.867	
Age	0.0224	0.0180	12.396	0.237	0.2734
Seller gender	0.1201	0.3753	0.3200	0.754	0.1038
Affective trustworthiness	0.5465	0.2406	22.711	0.041*	0.6135
Seller performance	0.0630	0.2663	0.2365	0.817	0.0506
Manipulatory communication	−0.0278	0.1064	−0.2610	0.798	−0.0416
AWI_InitialSection	0.2953	0.1313	22.502	0.042*	14.484
AWI_CentralSection	−0.4227	0.2027	−20.854	0.057	−15.289
AWI_FinalSection	0.1131	0.0773	14.625	0.167	0.3785
WPI_CentralSection	−0.7185	0.4779	−15.034	0.157	−25.476
WPI_InitialSection	0.2439	0.1571	15.530	0.144	12.351
WPI_FinalSection	0.3743	0.2679	13.971	0.186	14.298
BAR_InitialSection	−169.663	69.564	−24.389	0.030*	−0.7175
BAR_CentralSection	76.170	206.258	0.3693	0.718	0.5569
BAR_FinalSection	−0.7114	226.734	−0.0314	0.975	−0.0440
BATR_InitialSection	74.682	38.594	19.351	0.075	0.5515
BATR_CentraleSection	−198.231	266.031	−0.7451	0.469	−16.916
BATR_FinalSection	125.485	279.759	0.4485	0.661	0.9704

(C) Assumption checks			
Test type		Statistic	*p*
Normality test	Shapiro–Wilk	0.978	0.743
Heteroskedasticity test	Breusch-Pagan	11.5	0.78

**(A)** Model fit measures table, **(B)** Model coefficients table. *p < 0.05, **(C)** Assumption checks table.

A third linear regression model was conducted to observe which of the investigated variables had a significant impact on Spot Likeability (Table 3). The regression model accounts for 65% of the variance (Adjusted *R*^2^ = 0.646, *F* = 4.014, *p* = 0.006) (Table 3A). Specifically, it suggests that the Neurophysiological indicator BATR recorded in the Initial sections of the spot is the only one significantly explaining the final infomercial Likeability response (*β*st = 0.644, *p* = 0.040) (Table 3B). Also here Normality and Heteroskedasticity assumptions were checked and found to be met (Table 3C).

**Table 3:** Linear regression model with infomercial Likeability as dependent variable.

(A) Model fit measures								
					Overall model test			
Model	*R*	*R* ^2^	Adjusted *R*^2^	AIC	*F*	df1	df2	*p*
1	0.923	0.851	0.646	91.4	4.14	18	13	0.006

(B) Model coefficients - infomercial likeability					
Predictor	Estimate	SE	*t*	*p*	Stand. estimate
Intercept	−0.7823	0.8972	−0.872	0.399	
Age	0.0222	0.0219	1.014	0.329	0.221
Seller gender	0.1444	0.4561	0.317	0.757	0.101
Affective trustworthiness	0.3806	0.2924	1.301	0.216	0.347
Seller performance	0.5442	0.3236	1.682	0.117	0.355
Manipulatory communication	0.1074	0.1294	0.830	0.421	0.131
AWI_InitialSection	0.2335	0.1595	1.464	0.167	0.930
AWI_CentralSection	−0.2908	0.2464	−1.180	0.259	−0.854
AWI_FinalSection	0.0534	0.0940	0.569	0.579	0.145
WPI_CentralSection	0.1947	0.5808	0.335	0.743	0.561
WPI_InitialSection	−0.1069	0.1909	−0.560	0.585	−0.440
WPI_FinalSection	−0.0856	0.3256	−0.263	0.797	−0.266
BAR_InitialSection	−163.284	84.547	−1.931	0.076	−0.561
BAR_CentralSection	119.666	250.683	0.477	0.641	0.711
BAR_FinalSection	−79.994	275.568	−0.290	0.776	−0.402
BATR_InitialSection	107.313	46.906	2.288	0.040*	0.644
BATR_CentraleSection	−182.697	323.329	−0.565	0.582	−1.266
BATR_FinalSection	113.198	340.015	0.333	0.745	0.711

**(C)** Assumption checks			
Test type		Statistic	*p*
Normality test	Shapiro–Wilk	0.953	0.18
Heteroskedasticity test	Breusch-Pagan	17	0.388

**(A)** Model fit measures table, **(B)** model coefficients table. *p < 0.05, **(C)** assumption check table.

### 4.2. Eye-tracking results

The analysis of participants’ perceptual-exploratory and attentional behaviors, carried out through eye-tracking technology, shows interesting correlations between the Time Spent (TS) on certain Areas Of Interest (AOIs) crucial to the infomercial, and the neurophysiological indicators (Table 4).

**Table 4:** Pearson correlations matrix performed between eye-tracker data (Time spent) and EEG metrics.

		TimeSpent Logo AOI	TimeSpent Discount AOI	TimeSpent Price AOI
AWI	Pearson’s *r*	0.404*	0.039	−0.352
	df	38	28	24
	Value of *p*	0.010	0.839	0.078
WPI	Pearson’s *r*	0.420*	0.605***	0.409*
	df	26	31	27
	Value of *p*	0.026	<0.001	0.028
BATR	Pearson’s *r*	−0.342	−0.035	0.112
	df	28	31	27
	Value of *p*	0.064	0.847	0.562
BAR	Pearson’s *r*	−0.309	−0.093	0.055
	df	28	31	27
	Value of *p*	0.096	0.607	0.776

*p < 0.05. ***p < 0.001.

Specifically, a positive Correlation is shown between TimeSpent on the brand Logo present for the whole stimulus duration and AWI [Pearson’s *r* = 0.404(38), *p* = 0.010] and WPI [Pearson’s *r* = 0.42(26), *p* = 0.026]; TimeSpent on the Discount and WPI registered in the specific frames in which the Discount AOI was present [Pearson’s *r* = 0.605(31), *p* < 0.001]; TimeSpent on the Price and the WPI registered in the specific frames in which the Price AOI was present [Pearson’s *r* = 0.409(27), *p* = 0.028]. These results can all be seen in Table 4. The more time these crucial areas are observed, the more it will increase our Willingness to Pay and our Interest and willingness to approach the infomercial and sponsored product.

## 5. Discussion

This study tried to understand the impact of some variables in the selling process through neuromarketing techniques. Tools used by neuromarketing, indeed, allow to capture the emotional reactions and dynamics occurring when subjects are stimulated and are involved in a decision process. As mentioned, neuroscience tools can be applied in any communication exchange activating a detectable and observable reaction (Plassmann et al., 2015; Bazzani et al., 2020).

Selling is not deeply explored from this point of view and neuroscience can be a key factor to detect the reactions of consumers. This is particularly crucial considering that consumers use mainly three areas of the brain, related to visual, emotional, and rational responses (Hill and Simon, 2010). Therefore, detecting also the emotional and non-verbal aspects of the consumers’ responses is crucial and neuromarketing and consumer neuroscience tools can cover this important aspect. In this sense, the evaluation of the reactions can be effectively detected in real time through neuromarketing techniques without the mediation of the cognitive side. This is a key point since they give instruments to have different interpretations between emotions really experienced and the rational side, with the possibility to observe the processing of information considering the role played by emotions (Passyn and Sujan, 2006).

This study, therefore, applied neuroscience to selling in a neuroselling point of view, to understand reactions in the selling process while looking at teleshopping communication as literature described this as growing (Bouhlel, 2019), characterized by a high level of trust, credibility, persuasion and by several relational elements (Dibble et al., 2016; Muda and Hamzah, 2021). Understanding dynamics in such types of communication and the effect on consumers can contribute to understanding human reactions in this variable setting and, moreover, in optimizing sellers’ effectiveness and increasing sales from a business perspective.

As for data, among the performed analyses some interesting results emerged. Regression analyses suggested that subjects were pushed to call the number to obtain information about the infomercial, particularly by the seller performance and the WPI in the initial part of the teleshopping. These elements are crucial to allow consumers to get into a relationship with both the seller and the product. A seller that communicates credibility and makes effective persuasive communications, can be a precious source for selling success and consumer trust (Pornpitakpan, 2004; Muda and Hamzah, 2021). Moreover, WPI in the initial part has a role in enhancing Call-to-Action, whereas WPI in the central part has a role in decreasing Call to Action, maybe because of the too much information, and the repetitiveness of this part of the teleshopping which may have bored viewers. These results suggest the importance of monitoring information at the beginning of the viewed teleshopping as each phase can significantly contribute to the WPI and buying behavior as suggested by other researchers (Sahai et al., 2020; Russo et al., 2022).

Purchase Intention seems to be influenced by the affective side of trust in sellers, revealing not only the importance of trustworthiness when buying without tangible elements (as in the teleshopping), but also the relevance of the relationship emerging by the affective evaluation that consumers are doing while observing the seller. In particular in teleshopping, as highlighted by Kline (2005), trust and relationship create sense, believability, stimulate the audience and create consensus and engagement around the products. This is in line with the other data that can influence the Purchase Intention: AWI in the initial phase. As it has a motivational side and is linked to the approach to a stimulus, this significant data may suggest that relation in the first impact and approach is a crucial evaluative element. In this analysis, BAR has a negative effect in purchase intention again in the initial phase and this would be in line with the previous data: being in trust and in relationship with the seller have an effect on consumers and on the intention to approach the product, but the communication should not be excessively exciting. In order to promote the product and to be persuasive, the communication style should be professional and put at their ease the consumer through technical knowledge (Futrell, 2006).

Finally, the infomercial likeability seems to be influenced only by BATR in the initial phase of the viewed teleshopping. This is a crucial element considering that cognitive engagement can have an important role on the evaluation of a product, on buying behavior and even in the creation of a sort of brand or product love (Shin and Back, 2020).

Considering Eye-Tracker results, the Time Spent on the logo, on the Discount and on the Price show significant correlation with WPI. Only the logo shows a significant correlation with AWI. As WPI, the selected AOIs show the importance of the recognizable logo and of the clear and visible price and discount as crucial elements for the possibility of buying from the consumers. Not only, this measure is linked also to the evaluation of convenience that subjects can make thanks to the possibility of having clear information about the product, its costs, and characteristics (Russo et al., 2021). AWI is significantly correlated only with the logo AOI and this could be an element linked to trust: having clear information about the company while buying at a distance can be reassuring.

Gender does not produce different effects among the two infomercials. This aspect suggests a homogeneity between the two infomercials and a good narrative structure that vehiculates the information in an effective way considering the Time Spent on the selected AOI and data on Call-to-Action, Purchase Intention, and Infomercial Likeability.

This study highlights important elements in particular the relationship and trust that the seller can communicate while selling. Many elements can be monitored, such as the ability to communicate in a professional and emotional way. In particular, results on performance suggest the importance of monitoring and developing the seller’s performance. A contemporary and systematic review of the literature on the characteristics of effective salespeople found that personal, organizational, collaborative, buyer, and situational dimensions are responsible for increasing salespeople’s performance (Herjanto and Franklin, 2019). However, there is little exploration of the competencies of an effective sales representative in the existing literature (Ben Amor, 2019). Some recent research has examined the determinants of salesperson performance (Ahearne and Schillewaert, 2000; Johlke, 2006). An interesting work by Rentz et al. (2002) proposes three dimensions that have to be considered in sales skills: interpersonal skills, communication style skills, and technical skills. More specifically, interpersonal skills correspond to listening, empathy, optimism, and perceived observation skills. Studies have shown a significant and positive relationship between these sub-dimensions and performance (Rich and Smith, 2000; Rapisarda, 2002). Communication style skills refer to the ability to sell or persuade people to buy (adaptability, negotiation, and questioning, the salesperson’s communication style, and consultative selling). They are fundamental to making a sales presentation and closing the sale (Manning and Reece, 2004). Finally, technical skills correspond to the ability of salespeople to provide information about the design, specifications, applications, and functions of products or services (Churchill et al., 2000; Futrell, 2006). The use of technical knowledge induces higher salesperson performance. Monitoring these aspects can be important for both the consumers that have the possibility to face professional sellers, and the company for the sales success from a business perspective.

The study’s limitations are related to a small sample size and a focus on specific infomercials. Enlarging the sample size could be useful also to explore other effects that in this study were not significant or very close to significance, in particular in the closing part of the teleshopping. However, according to Jenkins and Quintana-Ascencio (2020), the sample size is in line with studies based on biological data and also with the recommendation for regression models of *N* = 8 with low variance, and a minimum of *N* = 25 for high variance, as in the present study. Nevertheless, as this is a first study on teleshopping, we argue that future research could explore other infomercial types and further examine seller communication styles to assess other identification or approach-withdrawal effects. Additionally, it’s important to dig into which specific aspects of seller performance could have the potential to enhance Purchase Intention and Call-to-Action. Future studies should consider more variables to undertake a more exhaustive exploration of seller performance, thereby uncovering the distinct dimensions of seller performance that could yield a positive impact on the overall infomercial success.

Moreover, we decided not to investigate whether the brand, salesperson, or adv was already known by the participants since we wanted to ensure that participant’s responses to the stimuli remained unaffected. In fact, topics like the salesperson or brand should be the focus of future research.

Finally, traditional techniques (questionnaires, interviews, and focus groups), can provide a variety of information about an emotional reaction, from a conscious perspective (Micu and Plummer, 2010). Neuroscientific measures, on the other hand, provide an output of emotional experience that is not mediated by cognitive processing (Poels and DeWitte, 2006) or distorted by cognitive bias (Haley et al., 1994). Indeed, sometimes people omit or alter their responses in order to safeguard their image and express what is considered most socially acceptable (Edwards, 1957; Merlowe and Crowne, 1961; Crosby et al., 1980; DeMaio, 1984; Paulhus, 1984; Maass et al., 2000; Roccato and Zogmaister, 2010). In addition, participants’ responses may be unintentionally distorted due to limitations in the ability to introspect (Greenwald and Banaji, 1995; Boca, 1996; Kitawaki and Hiromi, 1998; Banaji and Mahzarin, 2001) and difficulties in verbally expressing emotions (Penn, 2006). Therefore, the combination of the two approaches should be considered necessary and complementary. The lack of reliable approaches to anticipate consumer behavior can generate significant consequences. In fact, it is estimated that between 40 and 80% of new products introduced to the market are unsuccessful, causing economic damage to companies that translates into losses of billions of dollars (Castellion and Markham, 2012; Hakim and Levy, 2019). In the future, the integration of other traditional techniques side by side with neuroscientific ones may elucidate new and as yet little known aspects on the topic.

Despite its limitations, this project is a noteworthy pilot study that highlights crucial emotional aspects of the seller/buyer relationship that can affect sales decisions, relationships, and outcomes. Moreover, this study gives an important contribution to defining neuroselling as a discipline. Neuromarketing techniques give this project a novel and pioneering edge as they reveal unknown emotional reactions and improve understanding of factors affecting decision-making, leading to a reevaluation of communication dynamics for better business performance.

## Data availability statement

The raw data supporting the conclusions of this article will be made available by the authors, without undue reservation.

## Ethics statement

The studies involving humans were approved by IULM University Ethical Committee. The studies were conducted in accordance with the local legislation and institutional requirements. The participants provided their written informed consent to participate in this study.

## Author contributions

VR, MB, CC, SC, MC, AF, FR, CR, RV, and MZ contributed to the present study and wrote the manuscript. AF, MC, and SC designed the research. FR, CC, and CR collected the data and carried out data analysis and interpretation. RV and MB edited the final version. VR and MZ supervised the project and the manuscript writing. All authors have approved the final version of the manuscript and they are accountable for the whole work.
